# Supplementary material for: Postnatal oogenesis leads to an exceptionally large ovarian reserve in naked mole-rats
Source: Nat Commun. 2023 Feb 21;14:670. doi: 10.1038/s41467-023-36284-8 (PMC9944903; doi:10.1038/s41467-023-36284-8)
Supplement: Supplementary file 1 — Supplementary Information [file 41467_2023_36284_MOESM1_ESM.pdf]

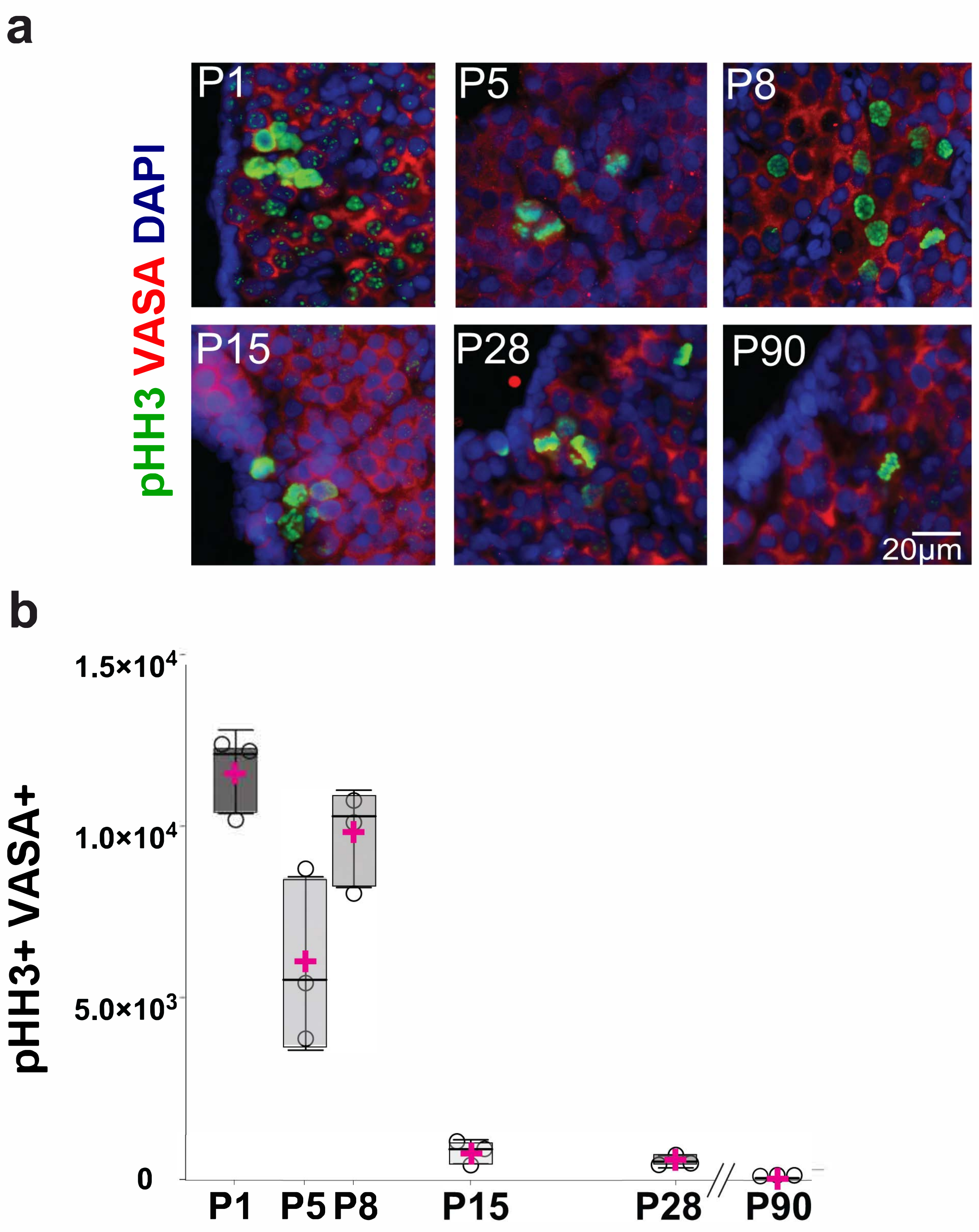

Supplementary Figure 1. a) In vivo postnatal germ cell expansion analyzed using immunofluorescence detection of the marker of mitotic cell division pHH3 (green) and the germ cell marker VASA (red). b) Quantification of the mitotically active germ cells along the different ages using the pHH3 and VASA as markers at P1, P5, P8, P15, P28, and P90 (n=3 per group). The box edges represent 25th and 75th percentiles, the horizontal line inside the box represents the median, and pink +' represents the mean. One-way-ANOVA, adjusting for multiple comparisons using the Bonferroni method, all pairwise comparisons were statistically significant at $p < 0.0001$, except for age 5 vs age 8 ($p = 0.10$), age 1 vs. age 8, age 15 vs. age 28, age 15 vs. age 90 and age 28 vs. age 90 (all $p > .99$) days. Source data are provided as a Source Data file.

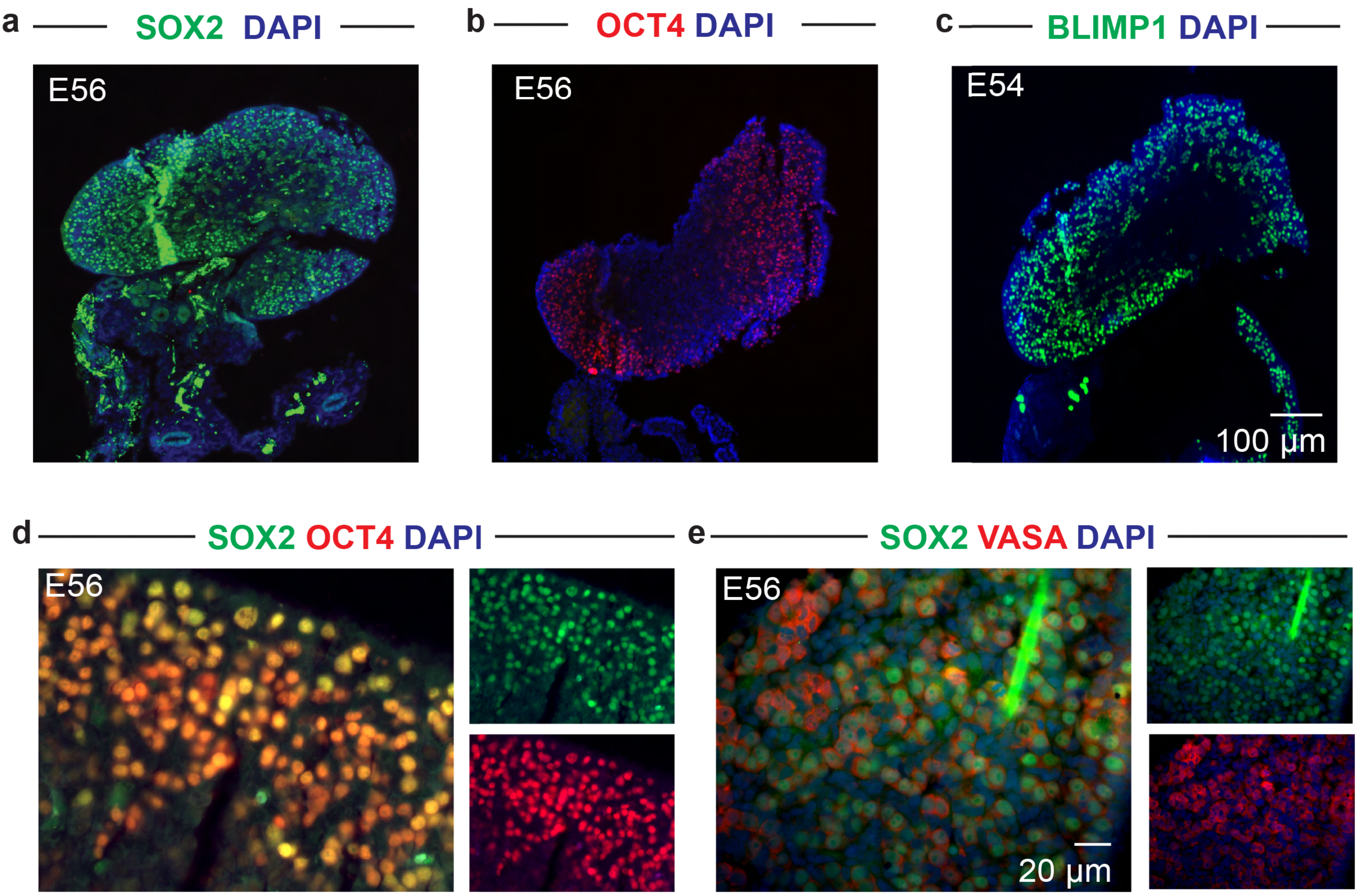

Supplementary Figure 2. IF for markers of pluripotency and primordial germ cells in fetal naked mole-rat (NMR) ovaries. a) Embryonic day 56 (E56) NMR ovary stained with antibody against pluripotency marker SOX2 (green) and DAPI (blue). b) E56 NMR ovary stained with antibody against pluripotency marker OCT4 (red) and DAPI (blue). c) E56 NMR ovary stained with antibody against pluripotency marker BLIMP1 (green) and DAPI. d) Co-localization of pluripotency markers SOX2 (green) and OCT4 (red). e) IF showing the presence of cells positive for the germ cell marker VASA (red), the pluripotency marker SOX2 (green) and DAPI (blue) in E56 NMR ovary.

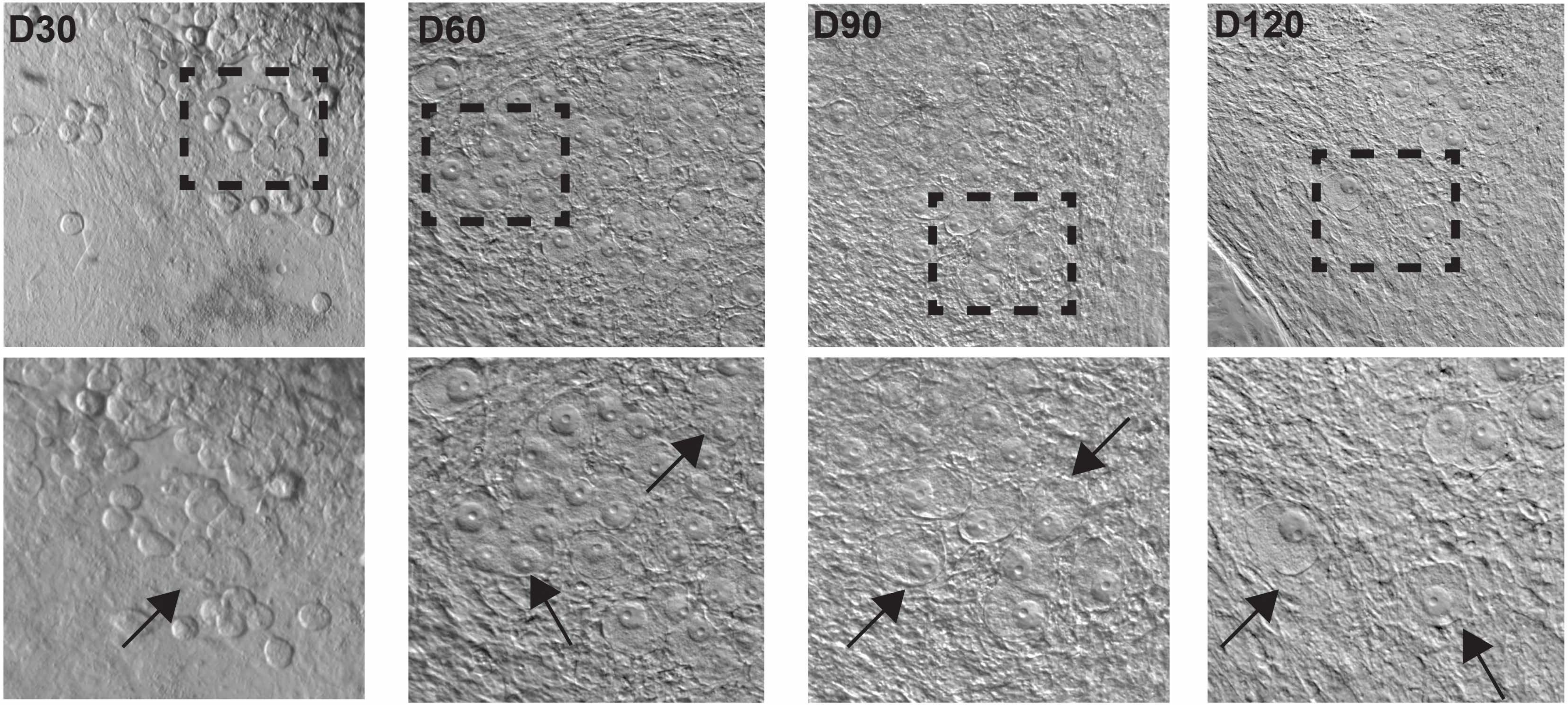

Supplementary Figure 3. In vitro expansion of germ cells from P5 ovaries. Representative images of cultures at 30 days of culture (D30) showing incip-ient germ cell nest; 60 days (D60) showing germ cell nests, primordial follicles; 90 days of culture (D90), showing primordial follicles and 120 days of culture (D120) showing primordial follicles.

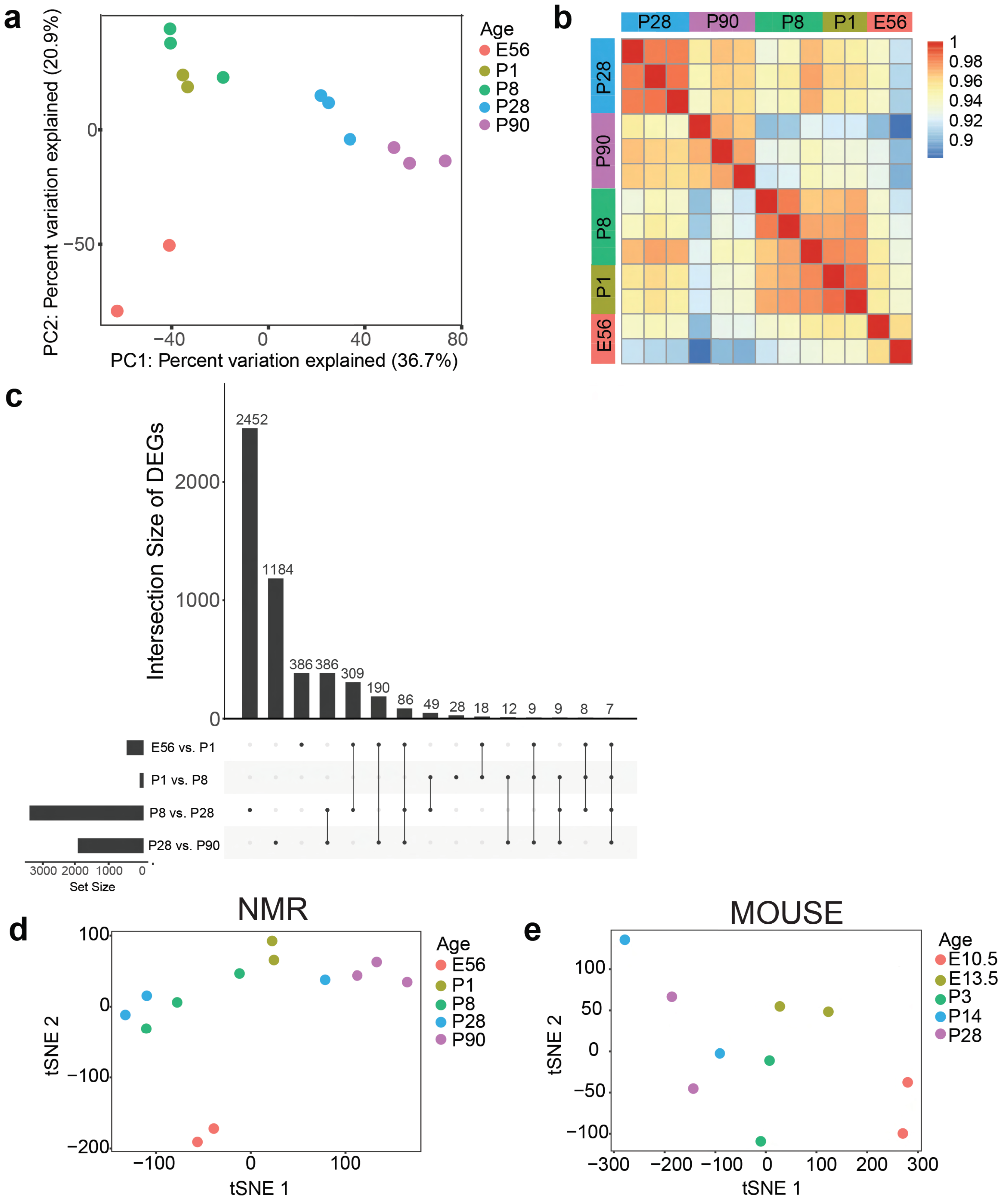

Supplementary Figure 4. Quality control for RNA sequencing processing. a) Principal component analysis showing the distribution of the samples analyzed (E56, P1, P8, P28, and P90; RLE-normalized log CPM counts) in the naked mole-rat ovary. b) Correlation matrix using RLE-normalized log CPM counts between samples. c) Upset plot was generated from the differentially-expressed genes (DEGs) of the four sequential between-ages comparisons. The number of significant DEGS (adjusted p-value < 0.05 and log2 fold-change > abs(log2(1.5)) per comparison are as follows: E56 vs. P1 = 462, P1 vs. P8 = 60, P8 vs. P28 = 3308, P28 vs. P90 = 1892. d) t-SNE plot showing the distribution of the samples analyzed (E56, P1, P8, P28, and P90; transcripts per million) in the naked mole-rat ovary. e) t-SNE plot showing the distribution of the samples analyzed (E10.5, E13.5, PND3, PND14, PND28; transcripts per million) in the mouse ovary. Data was re-processed from Cardoso-Moreira et al., 2019.

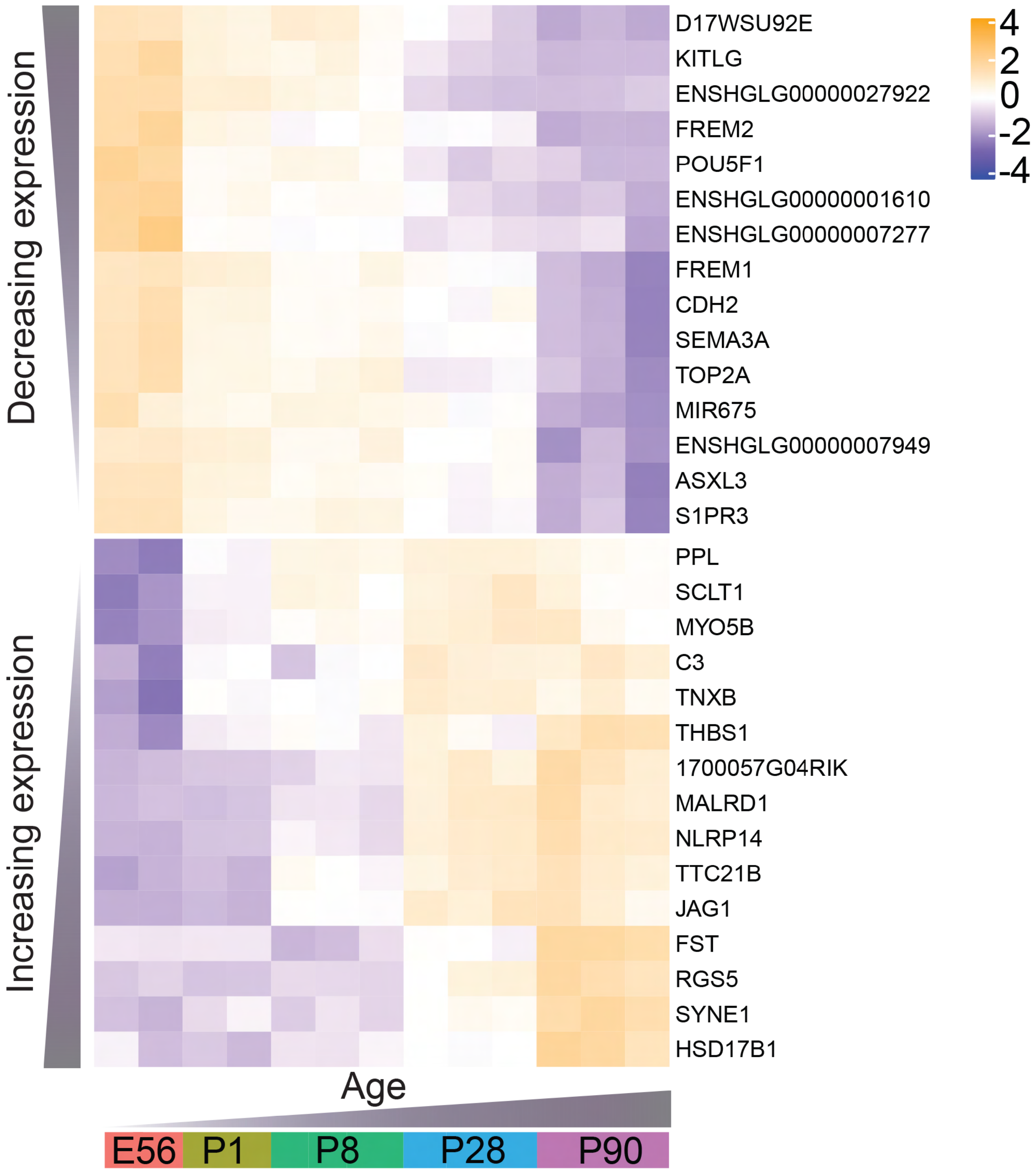

Supplementary Figure 5. A heatmap of top 10 genes that increase expression (counts per million) across ages and the top 10 genes that decrease expression across ages. Genes were identified using DESeq2 time-series analysis.

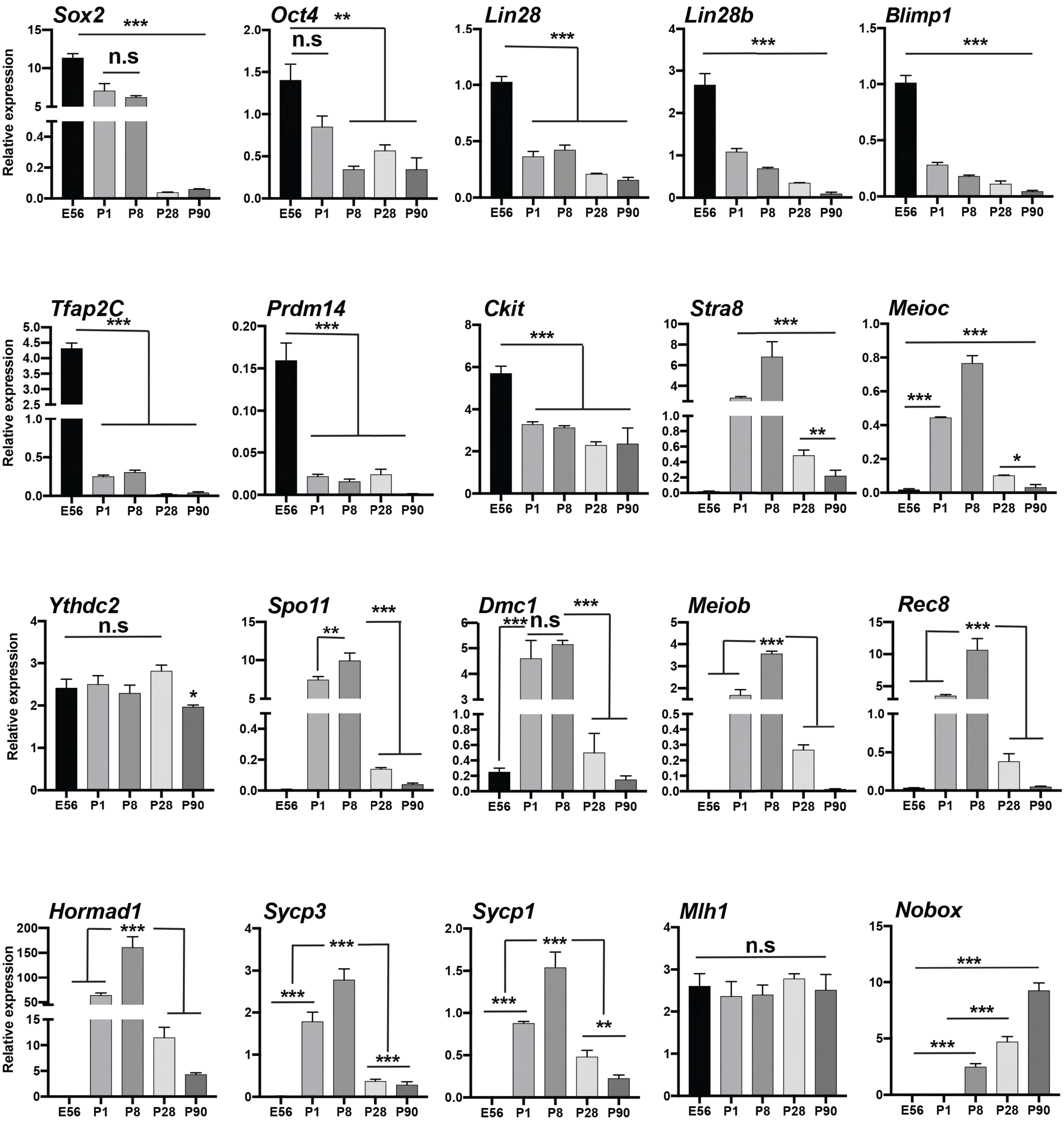

Supplementary Figure 6. Real-time qPCR analysis of the genes involved in pluripotency, germ cell development, meiosis commitment, double strand break formation, repair, and resolution, and folliculogenesis. Relative quantification versus housekeeping genes *Sdha* and *Mapk1*. Statistical analysis performed by two-way ANOVA, repeated measures, p=0.05. Source data are provided as a Source Data file.

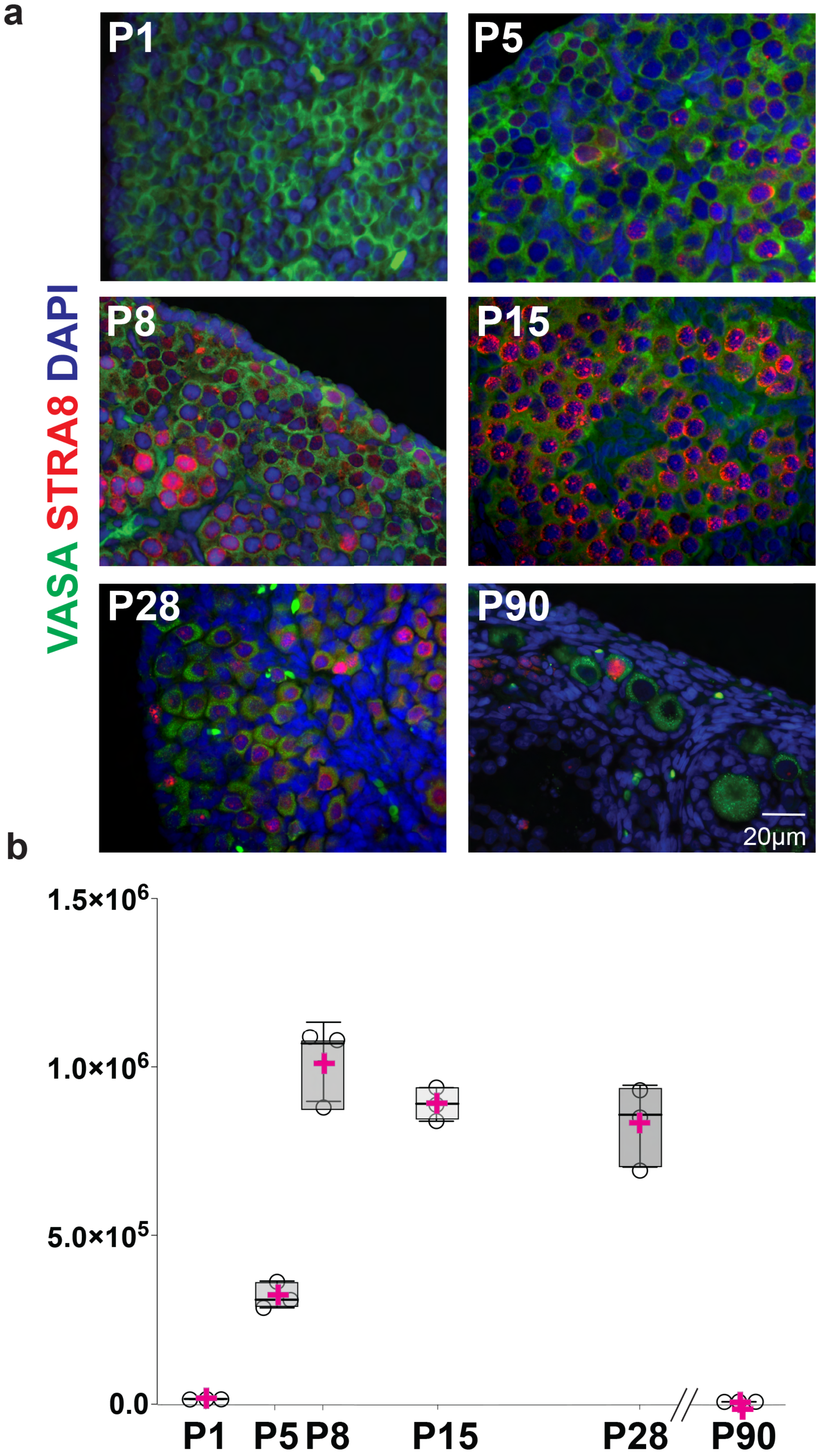

Supplementary Figure 7. Meiosis initiation program analyzed by Stimulated by Retinoic Acid Gene 8 Homolog (STRA8). a) immunolocalization among the different ages (P1, P5, P8, P15, P28 and P90), VASA (green), STRA8 (red) and DAPI (blue). b) Quantification of VASA+STRA8+ cells at P1, P5, P8, P15, P28 and P90 (n=3 per group). The box edges represent 25th and 75th percentiles, the horizontal line inside the box represents the median, and the pink'+' represents the mean. One-way-ANOVA, adjusting for multiple comparisons using the Bonferroni method, all pairwise comparisons were statistically significant at $p < 0.0001$, except for P90 vs P1 ($p > 0.99$).

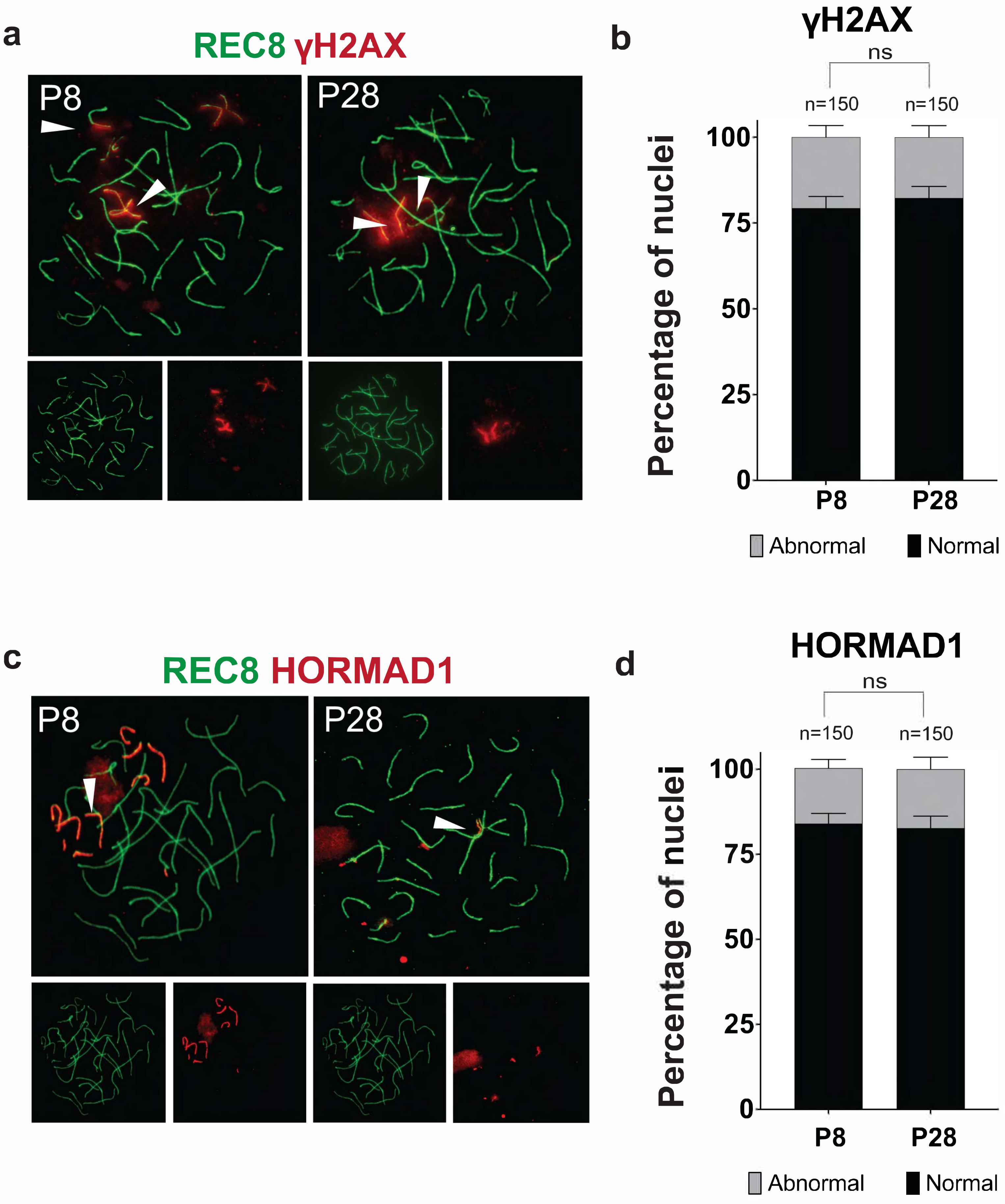

Supplementary Figure 8. Abnormal meiotic progression on NMR oocytes. a) Persistence of γH2AX (red) signal on chromosome spreads of oocytes at P8 and P28. White arrows indicate the places of the persistence. b) Percentages of nuclei showing retention of γH2AX (red) at P8 and P28, which were not statistically different. c) Asynapsed chromosome regions shown by the presence of HORMAD1 protein on oocytes from P8 and P28. White arrows indicate the areas of asynapsis. d) Percentages of nuclei showing retention of HORMAD1 (red) at P8 and P28, which were not statistically different.

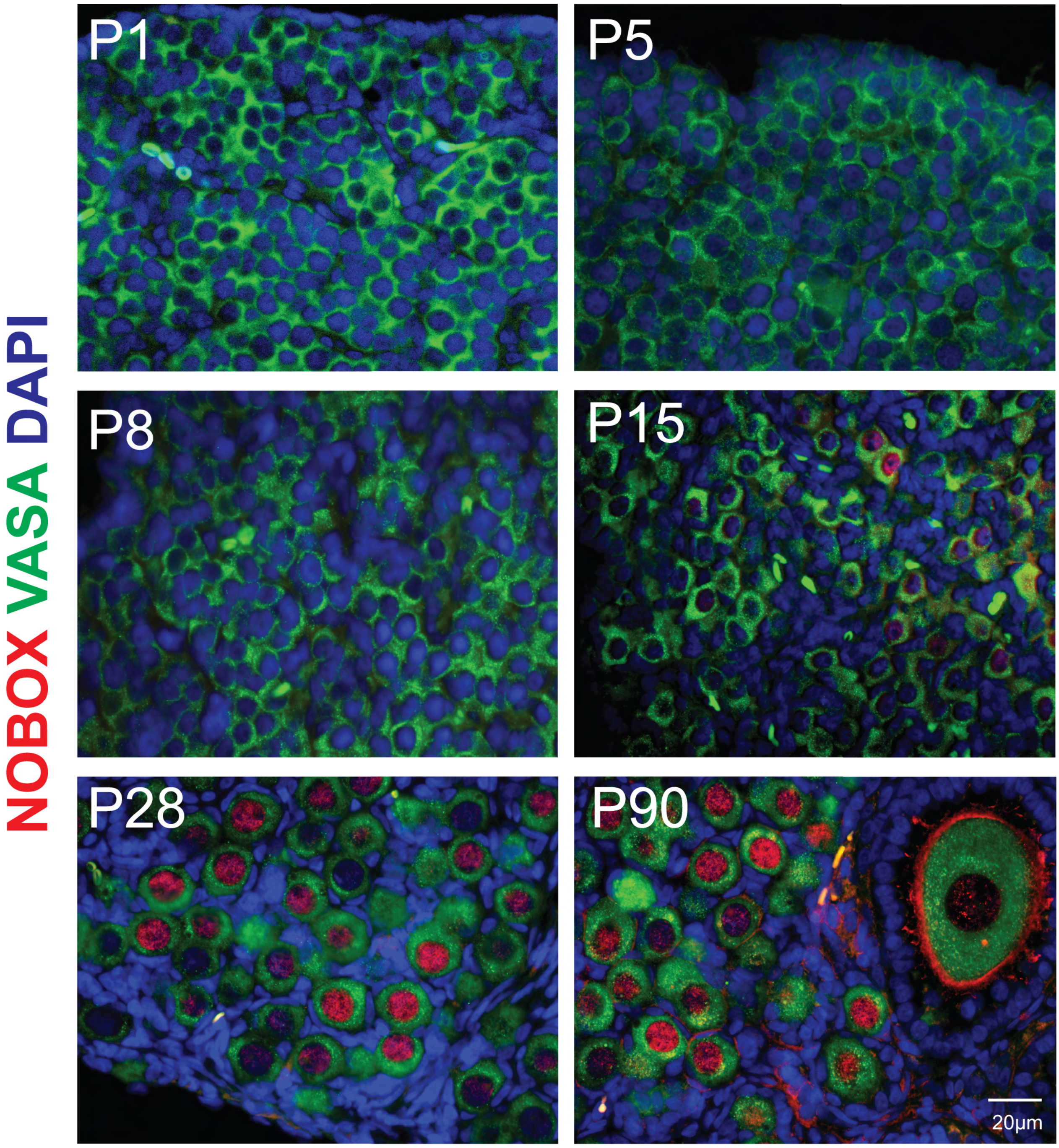

Supplementary Figure 9. Transition from primordial follicles to primary follicle. Transition from pre-follicle germ cells to primordial and primary follicles detected by the presence of NOBOX (red), VASA (green) and DAPI (blue) at P1, P5, P8, P15, P28 and P90.

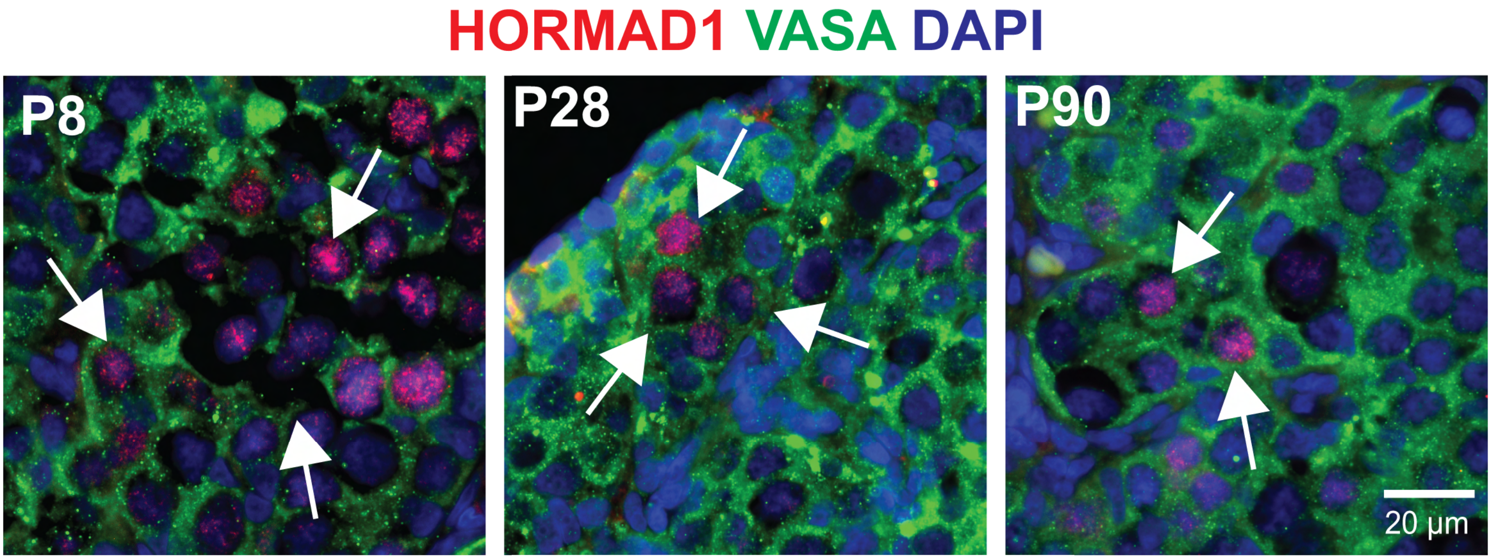

Supplementary Figure 10. Immunodetection of HORMAD1 on histological sections of NMRs at postnatal day 8(P8), 28 (P28) and 90 (P90). HORMAD1 (red), VASA (green) and DAP1 (blue).White arrows indicate representative HORMAD1+VASA+ cells.
