# Supplementary material for: Postnatal oogenesis leads to an exceptionally large ovarian reserve in naked mole-rats
Source: Nat Commun. 2023 Feb 21;14:670. doi: 10.1038/s41467-023-36284-8 (PMC9944903; doi:10.1038/s41467-023-36284-8)
Supplement: Supplementary file 3 — Description of Additional Supplementary Files [file 41467_2023_36284_MOESM3_ESM.pdf]

Description of Additional Files:

Supplementary Data File 1-16: RNA-seq comparisons

Supplementary Data File 1: Differentially expressed genes between E56 and P1 ovaries

Supplementary Data File 2: Differentially expressed genes between P1 and P8 ovaries.

Supplementary Data File 3: Differentially expressed genes between P8 and P28 ovaries.

Supplementary Data File 4: Differentially expressed genes between P28 and P90 ovaries.

Supplementary Data File 5: GSEA pathways that are downregulated between E56 and P1 8 ovaries.

Supplementary Data File 6: GSEA pathways that are upregulated between E56 and P1 ovaries.

Supplementary Data File 7: GSEA pathways that are downregulated between P1 and P8 ovaries.

Supplementary Data File 8: GSEA pathways that are upregulated between P1 and P8 ovaries.

Supplementary Data File 9: GSEA pathways that are downregulated between P8 and P28 ovaries.

Supplementary Data File 10: GSEA pathways that are upregulated between P8 and P28 ovaries.

Supplementary Data File 11: GSEA pathways that are downregulated between P28 and P90 ovaries.

Supplementary Data File 12: GSEA pathways that are upregulated between P28 and P90 ovaries.

Supplementary Data File 13: Differentially expressed time-series genes.

Supplementary Data File 14: GSEA pathways that are upregulated across time-series genes.

Supplementary Data File 15: GSEA pathways that are downregulated across time-series genes.

Supplementary Data File 16: Quality control metrics for RNA sequencing data

Supplementary Data File 17: Antibodies used

Supplementary Data File 18: Primers used

Supplementary Data File 19: Cell counts for each marker and time analyzed
