# Supplementary material for: Postnatal oogenesis leads to an exceptionally large ovarian reserve in naked mole-rats
Source: Nat Commun. 2023 Feb 21;14:670. doi: 10.1038/s41467-023-36284-8 (PMC9944903; doi:10.1038/s41467-023-36284-8)
Supplement: Supplementary file 8 — Reporting Summary [file 41467_2023_36284_MOESM8_ESM.pdf]

nature portfolio

Corresponding author(s): Miguel Angel Brieño-Enríquez
Ned J. Place

Last updated by author(s): 2023/01/10

Reporting Summary

Nature Portfolio wishes to improve the reproducibility of the work that we publish. This form provides structure for consistency and transparency in reporting. For further information on Nature Portfolio policies, see our Editorial Policies and the Editorial Policy Checklist.

Statistics

For all statistical analyses, confirm that the following items are present in the figure legend, table legend, main text, or Methods section.

n/a	Confirmed	
☐	☒	The exact sample size (*n*) for each experimental group/condition, given as a discrete number and unit of measurement
☐	☒	A statement on whether measurements were taken from distinct samples or whether the same sample was measured repeatedly
☐	☒	The statistical test(s) used AND whether they are one- or two-sided<br>*Only common tests should be described solely by name; describe more complex techniques in the Methods section.*
☒	☐	A description of all covariates tested
☐	☒	A description of any assumptions or corrections, such as tests of normality and adjustment for multiple comparisons
☐	☒	A full description of the statistical parameters including central tendency (e.g. means) or other basic estimates (e.g. regression coefficient) AND variation (e.g. standard deviation) or associated estimates of uncertainty (e.g. confidence intervals)
☐	☒	For null hypothesis testing, the test statistic (e.g. *F*, *t*, *r*) with confidence intervals, effect sizes, degrees of freedom and *P* value noted<br>*Give P values as exact values whenever suitable.*
☒	☐	For Bayesian analysis, information on the choice of priors and Markov chain Monte Carlo settings
☐	☒	For hierarchical and complex designs, identification of the appropriate level for tests and full reporting of outcomes
☒	☐	Estimates of effect sizes (e.g. Cohen's *d*, Pearson's *r*), indicating how they were calculated

Our web collection on statistics for biologists contains articles on many of the points above.

Software and code

Policy information about availability of computer code

Data collection	Image acquisition was performed using a Zeiss AxioImager M2 microscope (Carl Zeiss AG, Oberkochen. Germany). Images were processed using Zen 2 (Carl Zeiss AG, Oberkochen. Germany). Cell counter feature in Fiji (Image J, NIH version12.3).
Data analysis	Read quality of the raw library was assessed using FastQC (v0.11.8) Trimming of adapters was performed using cutadapt (v1.8) Trimmed reads were mapped to the naked mole-rat genome using RSEM (v1.2.22) and STAR aligner (v2.6.0). The NMR genome and transcriptome annotation were taken from the ENSEMBL database (release 100; ftp://ftp.ensembl.org/pub/release-100/fasta/). All downstream analyses were performed in R (v4.0). Heatmaps were made using ComplexHeatmap (v 2.13.1). RSEM counts were imported with tximport (1.0.3). Pairwise differential expression on sequential ages was performed with a likelihood ratio test in DESeq2 (v1.38.2) using counts per million. Gene set enrichment for the time-series genes was performed using GSEA (v4.2.3). The fastq files for the mouse ovarian RNAseq data for E10.5, E13.5, P3, P14 and P28 were downloaded from https://www.ebi.ac.uk/arrayexpress/experiments/E-MTAB-6798/11. The data were processed using the same pipeline as the NMR RNAseq data, with the exception of using the mouse genome and transcriptome annotation (Mus musculus, ftp://ftp.ensembl.org/pub/release-100/fasta/; GENCODE M25). t-SNE plots for the NMR and mouse RNAseq data were then plotted using the t-SNE wrapper function in M3C (v1.20.0) All data were analyzed with the CFX-manager Bio-Rad, CFX Maestro Software 2.0 (Bio-Rad Laboratories).

For manuscripts utilizing custom algorithms or software that are central to the research but not yet described in published literature, software must be made available to editors and reviewers. We strongly encourage code deposition in a community repository (e.g. GitHub). See the Nature Portfolio guidelines for submitting code & software for further information.

Data

Policy information about availability of data

All manuscripts must include a data availability statement. This statement should provide the following information, where applicable:

The RNAseq data sets generated for this study are deposited in Gene Expression Omnibus (GEO) repository, under the accession number GSE139515. The mouse RNAseq data re-analyzed for this study can be found at https://www.ebi.ac.uk/arrayexpress/experiments/E-MTAB-6798/.
he NMR genome and transcriptome annotation were taken from the ENSEMBL database (release 100; ftp://ftp.ensembl.org/pub/release-100/fasta/). All downstream analyses were performed in R (v4.0).

Human research participants

Policy information about studies involving human research participants and Sex and Gender in Research.

Reporting on sex and gender	N/A
Population characteristics	
Recruitment	
Ethics oversight	*Identify the organization(s) that approved the study protocol.*

Note that full information on the approval of the study protocol must also be provided in the manuscript.

Field-specific reporting

Please select the one below that is the best fit for your research. If you are not sure, read the appropriate sections before making your selection.

X Life sciences ☐ Behavioural & social sciences ☐ Ecological, evolutionary & environmental sciences

Life sciences study design

All studies must disclose on these points even when the disclosure is negative.

Sample size	The number of NMRs required is based on one previous publication (Place et al., 2021) and power analyses to ensure detection of statistical significance. ($p=0.05$). Sample size was calculated using G*Power 3.1 45, with the following parameters: statistical test, ANOVA repeated measures, within-between interaction; effect size f: 0.25; α error probability: 0.05; power (1-ß err prob) 0.95. Sample size includes 15% subject attrition.
Data exclusions	*Three different ovaries were analyzed at each time point (RNA seq), however, we excluded E56-13 and P1-2 as these samples suggested a non-ovarian gene expression pattern (all RNAseq data is available in GEO).*
Replication	*All the experiments were successfully performed at least as triplicates, and for the cell counts 6 animals were included per category. All the counts were performed by different users, once they had all the data, values were pooled. Animals were harvest from different mothers and times.*
Randomization	*All experiments related to cell counts, cultures, molecular biology, library preparation, and sequencing will be performed in parallel to avoid methodological bias. NMRs were randomly chosen for experimental groups (for each time point animals were taken from different colonies and allocated in different groups randomly) , and multiple smaller replication cohorts were run to mitigate experimenter bias, day-to-day variability, and other factors arising from extraneous circumstances.*
Blinding	*All the experiments were performed by at least three different researchers to avoid bias (cell counts, cultures, qpcrs, etc.). Blinding was not possible as experimental conditions were evident from the image data. Quantifications were performed by three different researchers at different locations and then data was pooled.*

Behavioural & social sciences study design

All studies must disclose on these points even when the disclosure is negative.

Study description	
Research sample	
Sampling strategy	

Data collection

Timing

Data exclusions

Non-participation

Randomization

Ecological, evolutionary & environmental sciences study design

All studies must disclose on these points even when the disclosure is negative.

Study description

Research sample

Sampling strategy

Data collection

Timing and spatial scale

Data exclusions

Reproducibility

Randomization

Blinding

Did the study involve field work? ☐ Yes ☒ No

Field work, collection and transport

Field conditions *NA.*

Location *NA*

Access & import/export *NA*

Disturbance *NA*

Reporting for specific materials, systems and methods

We require information from authors about some types of materials, experimental systems and methods used in many studies. Here, indicate whether each material, system or method listed is relevant to your study. If you are not sure if a list item applies to your research, read the appropriate section before selecting a response.

Materials & experimental systems

n/a	Involved in the study
☐	X Antibodies
☐	X Eukaryotic cell lines
X	☐ Palaeontology and archaeology
☐	X Animals and other organisms
X	☐ Clinical data
X	☐ Dual use research of concern

Methods

n/a	Involved in the study
X	☐ ChIP-seq
X	☐ Flow cytometry
X	☐ MRI-based neuroimaging

Antibodies

Antibodies used

ANTI-HUMAN MLH1 (BD PHARMIGEN 550838); ANTI-GAMMA H2AX (S139) (ABCAM AB11174); ANTI-GAMMA H2AX (S139)(MILLIPORE 05-636-I); ANTI-SCP1(ABCAM AB15090); DDX4 (ABCAM AB13840); DDX4(ABCAM AB27591) STRA8 (ABCAM AB49602); CLEAVED PARP1 (ABCAM); KI67(ABCAM AB15580); OCT4(ABCAMAB19857); SOX2 (THERMOFISHER SCIENTIFIC MA1-014); pHH3 (S10) (ACTIVE MOTIF/THERMOFISHER SCIENTIFIC 61624 (6G8B7)); pHH3 (S10)(ABCAM AB14955); CLEAVED CASPASE3 (CELL SIGNALING 9661); CLEAVED CASPASE9 (CELL SIGNALING 20750); REC8 (JOSE LUIS BARBERO); NOBOX (ALEXANDER RAJKOVIC); HORMAD1 (ATTILA TOTH)

Validation

All antibodies were previously validated in NMR IPSCs and with the cell lines that we generated from primary cultures in the lab (kidney, heart, ovary, PGCs, fibroblast) For this purpose, we have already created different naked mole-rat cell lines obtained from primary cultures of fibroblast, kidney, heart, skin, and lung.

We developed new techniques that allowed us to culture primordial germ cells and expand their population in vitro. For IHC validation, these cell lines can be formalin-fixedlparaffin-embedded and serve as positive and negative controls.

Eukaryotic cell lines

Policy information about cell lines and Sex and Gender in Research

Cell line source(s)

Cell lines were generated from primary cultures at the lab

Authentication

Authentication was performed with IF, PCR

Mycoplasma contamination

NA

Commonly misidentified lines
(See ICLAC register)

NA

Palaeontology and Archaeology

Specimen provenance

Specimen deposition

Dating methods

☐ Tick this box to confirm that the raw and calibrated dates are available in the paper or in Supplementary Information.

Ethics oversight

Note that full information on the approval of the study protocol must also be provided in the manuscript.

Animals and other research organisms

Policy information about studies involving animals; ARRIVE guidelines recommended for reporting animal research, and Sex and Gender in Research

Laboratory animals

Naked Mole Rat (Heterocephalus glaber) at postnatal day 1, 5, 8, 15, 28, 3 and 6 month-old, and 3, 6 and 10 years-old.

Wild animals	*No wild animals were used in this study*
Reporting on sex	*Ovarian biology was the focus of this proposal, so our samples are sex restricted. A total of 50 NMR were used in this study*
Field-collected samples	*No field collected samples were used in this study*
Ethics oversight	*All experimental procedures followed federal and institutional guidelines and were approved by the Magee-Womens Research Institute, University of Pittsburgh (IACUC protocol #20117234) and University of Toronto Mississauga Animal Care and Use Committees (IACUC protocol # 20011632).*

Note that full information on the approval of the study protocol must also be provided in the manuscript.

Clinical data

Policy information about clinical studies

All manuscripts should comply with the ICMJE guidelines for publication of clinical research and a completed CONSORT checklist must be included with all submissions.

Clinical trial registration	*NA.*
Study protocol	*NA*
Data collection	*NA*
Outcomes	*NA*

Dual use research of concern

Policy information about dual use research of concern

Hazards

Could the accidental, deliberate or reckless misuse of agents or technologies generated in the work, or the application of information presented in the manuscript, pose a threat to:

No	Yes	
X	☐	Public health
X	☐	National security
X	☐	Crops and/or livestock
X	☐	Ecosystems
X	☐	Any other significant area

Experiments of concern

Does the work involve any of these experiments of concern:

No	Yes	
X	☐	Demonstrate how to render a vaccine ineffective
X	☐	Confer resistance to therapeutically useful antibiotics or antiviral agents
X	☐	Enhance the virulence of a pathogen or render a nonpathogen virulent
X	☐	Increase transmissibility of a pathogen
X	☐	Alter the host range of a pathogen
X	☐	Enable evasion of diagnostic/detection modalities
X	☐	Enable the weaponization of a biological agent or toxin
X	☐	Any other potentially harmful combination of experiments and agents

ChIP-seq

Data deposition

☐ Confirm that both raw and final processed data have been deposited in a public database such as GEO.

☐ Confirm that you have deposited or provided access to graph files (e.g. BED files) for the called peaks.

Data access links
May remain private before publication.

Files in database submission

Genome browser session
(e.g. UCSC)

Methodology

Replicates

Sequencing depth

Antibodies

Peak calling parameters

Data quality

Software

Flow Cytometry

Plots

Confirm that:

☐ The axis labels state the marker and fluorochrome used (e.g. CD4-FITC).

☐ The axis scales are clearly visible. Include numbers along axes only for bottom left plot of group (a 'group' is an analysis of identical markers).

☐ All plots are contour plots with outliers or pseudocolor plots.

☐ A numerical value for number of cells or percentage (with statistics) is provided.

Methodology

Sample preparation

Instrument

Software

Cell population abundance

Gating strategy

☐ Tick this box to confirm that a figure exemplifying the gating strategy is provided in the Supplementary Information.

Magnetic resonance imaging

Experimental design

Design type

Design specifications

Behavioral performance measures

Acquisition

Imaging type(s)

Field strength

Sequence & imaging parameters

Area of acquisition

Diffusion MRI ☐ Used ☐ Not used

Preprocessing

Preprocessing software

Normalization

Normalization template

Noise and artifact removal

Volume censoring

Statistical modeling & inference

Model type and settings — *Specify type (mass univariate, multivariate, RSA, predictive, etc.) and describe essential details of the model at the first and second levels (e.g. fixed, random or mixed effects; drift or auto-correlation).*

Effect(s) tested — *Define precise effect in terms of the task or stimulus conditions instead of psychological concepts and indicate whether ANOVA or factorial designs were used.*

Specify type of analysis: ☐ Whole brain ☐ ROI-based ☐ Both

Statistic type for inference (See Eklund et al. 2016) — *Specify voxel-wise or cluster-wise and report all relevant parameters for cluster-wise methods.*

Correction — *Describe the type of correction and how it is obtained for multiple comparisons (e.g. FWE, FDR, permutation or Monte Carlo).*

Models & analysis

n/a	Involved in the study
☐	☐ Functional and/or effective connectivity
☐	☐ Graph analysis
☐	☐ Multivariate modeling or predictive analysis

Functional and/or effective connectivity — *Report the measures of dependence used and the model details (e.g. Pearson correlation, partial correlation, mutual information).*

Graph analysis — *Report the dependent variable and connectivity measure, specifying weighted graph or binarized graph, subject- or group-level, and the global and/or node summaries used (e.g. clustering coefficient, efficiency, etc.).*

Multivariate modeling and predictive analysis — *Specify independent variables, features extraction and dimension reduction, model, training and evaluation metrics.*
